# Development and validation of a novel diagnostic model for initially clinical diagnosed gastrointestinal stromal tumors using an extreme gradient-boosting machine

**DOI:** 10.1186/s12876-021-02048-1

**Published:** 2021-12-18

**Authors:** Bozhi Hu, Chao Wang, Kewei Jiang, Zhanlong Shen, Xiaodong Yang, Mujun Yin, Bin Liang, Qiwei Xie, Yingjiang Ye, Zhidong Gao

**Affiliations:** Department of Gastrointestinal Surgery, Peking University People’s Hospital, No.11 Xizhimen South Street, Xicheng District, Beijing, 100044 China

**Keywords:** Gastrointestinal stromal tumor, Diagnostic model, XGBoost

## Abstract

**Introduction:**

Gastrointestinal stromal tumor (GIST) is the most common gastrointestinal soft tissue tumor. Clinical diagnosis mainly relies on enhanced CT, endoscopy and endoscopic ultrasound (EUS), but the misdiagnosis rate is still high without fine needle aspiration biopsy. We aim to develop a novel diagnostic model by analyzing the preoperative data of the patients.

**Methods:**

We used the data of patients who were initially diagnosed as gastric GIST and underwent partial gastrectomy. The patients were randomly divided into training dataset and test dataset at a ratio of 3 to 1. After pre-experimental screening, max depth = 2, eta = 0.1, gamma = 0.5, and nrounds = 200 were defined as the best parameters, and in this way we developed the initial extreme gradient-boosting (XGBoost) model. Based on the importance of the features in the initial model, we improved the model by excluding the hematological features. In this way we obtained the final XGBoost model and underwent validation using the test dataset.

**Results:**

In the initial XGBoost model, we found that the hematological indicators (including inflammation and nutritional indicators) examined before the surgery had little effect on the outcome, so we subsequently excluded the hematological indicators. Similarly, we also screened the features from enhanced CT and ultrasound gastroscopy, and finally determined the 6 most important predictors for GIST diagnosis, including the ratio of long and short diameter under CT, the CT value of the tumor, the enhancement of the tumor in arterial period and venous period, existence of liquid area and calcific area inside the tumor under EUS. Round or round-like tumors with a CT value of around 30 (25–37) and delayed enhancement, as well as liquid but not calcific area inside the tumor best indicate the diagnosis of GIST.

**Conclusions:**

We developed a model to further differential diagnose GIST from other tumors in initially clinical diagnosed gastric GIST patients by analyzing the results of clinical examinations that most patients should have completed before surgical resection.

**Supplementary Information:**

The online version contains supplementary material available at 10.1186/s12876-021-02048-1.

## Background

Gastrointestinal stromal tumors (GIST) are the most common gastrointestinal soft tissue tumors, accounting for about 20% of all soft tissue tumors [1]. GIST mainly occurs in the stomach, followed by the small intestine and colon, and rarely occurs in tissues outside the gastrointestinal tract (GI-tract) [2]. The clinical diagnosis of GISTs relies on enhanced computed tomography (CT), endoscopy and endoscopic ultrasound (EUS), but relying on these tests alone, or even their combination, has a high misdiagnosis rate. Moreover, GISTs are difficult to be differential diagnosed from other gastrointestinal submucosal tumors (SMTs) without fine needle aspiration biopsy [3, 4]. The pathology and immunohistochemistry results after fine needle aspiration biopsy are the most accurate ways to diagnose GISTs before surgery. Even though recent studies have confirmed that fine-needle aspiration biopsy will not result in tumor rupture or gastrointestinal dissemination of GISTs, it is still not frequently used in China and some other regions around the world [5, 6]. In addition, fine-needle aspiration has a certain false-negative rate because of the small specimen size, and patients often refuse to be examined because of the concerns about this invasive procedure. We still need a more convenient and non-invasive method to differential diagnose GISTs from other SMTs.

GISTs have a higher malignant biological potential compared with other gastrointestinal SMTs. According to the current GIST risk assessment standards and recommendations from guidelines around the world, most GISTs should undergo complete surgical resections [7]. For other SMTs that occur in the gastrointestinal tract, especially stomach, surgical intervention is generally not required. Therefore, creating a convenient and non-invasive model for further differential diagnosis of GISTs utilizing common examination results is pivotal to improving patients' quality of life and relieving economic stress.

The aim of this study is to develop a novel diagnostic model by analyzing the preoperative enhanced CT, endoscopy, EUS, and hematological test data of the patients. For this purpose, we trained an extreme gradient-boosting (XGBoost) model on a single-center dataset to predict the patient’s diagnosis, give the most important predictors and how they affect the predictive outcome [8].

## Methods

### Data

We retrospectively collected the data of all the patients who were initially diagnosed as gastric “gastrointestinal stromal tumor”, in which circumstances the patients with other SMTs that can be easily differentiated from GIST after CT scan or endoscopy were excluded, and completed surgical resection through January 2017 to June 2021 in the electronic medical record system of the Department of Gastrointestinal Surgery, Peking University People's Hospital. 128 continuous patients were screened. Among them, 4 patients were excluded due to the following reasons: 1 patient was accompanied by gastric cancer, 3 patients were missing important data in our electronic medical record system, because they had already performed preoperative examinations in other hospitals before admission. The remaining 124 patients were included in this study. We divided the patients into “GIST” and “other SMT” groups for comparison according to their postoperative pathological results. The t-test was conducted to compare continuous variables, as well as the Chi-square or Fisher’s test was utilized to compare categorical variables. Subsequently, we randomly divided all 124 patients into training dataset and validation dataset at a ratio of 3 to 1. The patient's enhanced CT, endoscopy, EUS, and hematological test results were included in this study, at least in initial model development. Missing values accounted for 12.78% of the total dataset. All statistical analysis, model training, validation and graphic plotting were based on R statistical software v4.0.3.

### Predictors

All 22 clinical test results potentially related to the patient's diagnosis were included in this study. All predictors are from enhanced CT, endoscopy, EUS, and hematological test results. Predictors from enhanced CT include tumor boundary, ratio of long to short diameter, homogeneity of enhancement, and the CT values of the tumor in different phases. Predictors from endoscopy include the existence of ulcers and bleeding on the mucosal surface of the tumor. Predictors from EUS include echogenicity, and the existence of calcification or liquid area inside the tumor. Besides, previous studies have suggested that some blood inflammatory and nutritious indicators are independent factors influencing the prognosis of gastrointestinal stromal tumors [9]. Therefore, we also tentatively included them as diagnostic predictors in this study, including peripheral blood leukocytes, neutrophils, lymphocytes, platelets, hemoglobin, alanine transaminase (ALT), glutamic oxaloacetic transaminase (AST), albumin levels, platelet–lymphocyte ratio (PLR), neutrophillymphocyte ratio (NLR), systemic immune-inflammation index (SII), De Ritis ratio (AST/ALT), prognostic nutritional index (PNI), and fibrinogen level. Data of all the hematological indicators were collected from the first test result after admission to our hospital and consideration for gastric GIST. The final diagnosis was based on the results of postoperative pathology and immunohistochemistry.

### Initial model development and selection of the predictors

Missing values were patched using the *missForest* package [10]. Then we incorporated all the 22 factors into the initial XGBoost model. After pre-experimental screening, max depth = 2, eta = 0.01, gamma = 0.25, and nrounds = 200 were defined as the best parameters and in this way we developed the initial XGBoost model. The *xgb.importance()* function of the *xgboost* package was employed to calculate the importance of each feature. After repeating the random grouping and model development 200 times, we can get the importance distribution of all 22 factors. Using the *ggplot* package to draw the box plot of their importance, we can see that the hematology test data generally has a very weak effect on the outcome. Therefore, we excluded the hematology test data in subsequent model development.

Using the same method for the second model development, it can be seen that three factors (the existence of ulcers on the mucosal surface of the tumor under gastroscopy, or bleeding, and homogeneity of enhancement in enhanced CT) also have a weak influence on the outcome, in which reason they were also excluded. The remaining 6 factors will be used to build the final model, including the ratio of long and short diameter under CT, the CT value of the tumor, the enhancement of the tumor in arterial period and venous period, existence of liquid area and calcific area inside the tumor under EUS.

### Final model development

Six most important predictors were selected using the previously described method, and checked for correlation by the *corrgram* package. The *xgboost* package was again applied to construct the XGBoost model in a similar way.

### Explain the model

The *xgb.importance()* function of the *xgboost* package was employed to calculate the importance of these 6 predictors and *ggplot2* package was employed to draw the box plot of the distribution of importance. Then we used the *explain()* function and *variable_effect()* function of the *DELAX* package to further elaborate how each predictor in the model affected the diagnostic outcome. The *predict_parts_break_down()* function was applied to illustrate the impact of these predictors on the diagnostic outcome in individual cases.

### Validation of the model

The *optimalCutoff()* function in the *InformationValue* package was used to obtain the best cutoff value of the model. The accuracy, precision, recall and f1-score of the model were then acquired from the test dataset, based on the given cutoff value. The receiver operating characteristic (ROC) curve was plotted with *plotROC()* function, and the area-under-ROC (auROC) value was subsequently calculated. The concordance index (C-index) was calculated with *rcorr.cens()* in the *Hmisc* package.

The final model development process was also repeated 200 times to acquire the distribution range of the predictor importance and evaluating results, and reported the 2.5 and 97.5 percentiles as 95 CI.

## Results

### Data description

A total of 124 patients who were initially diagnosed with gastric GISTs and underwent surgery from January 2017 to June 2021 were included in this study. 90 individuals were diagnosed as GIST according to postoperative pathology and immunohistochemistry. The other 34 patients were diagnosed as other SMT based on postoperative pathological diagnosis, including leiomyoma (n = 9), schwannoma (n = 5), lymphoma (n = 4), ectopic pancreas (n = 4), neurofibromas (n = 4), gastric duplication (n = 3), neuroendocrine tumors (n = 1), cysts (n = 1), congenital accessory spleen (n = 1), myofibroblastoma (n = 1), and lipoleiomyosarcoma (n = 1). 106 patients completed endoscopic examinations, all of which suggested the presence of submucosal lesions, and 62 of them had mucosal pathological biopsy taken under endoscopy. None of the patient’s preoperative mucosal biopsy detected tumors. 59 patients completed the preoperative EUS, and all showed submucosal mid-hypoechoic or hypoechoic lesions. Only 2 patients completed fine-needle aspiration biopsy, and the pathology showed gastrointestinal stromal tumors. All patients completed hematological test before surgery. All characteristics included in the initial analysis were shown in Table 1.

**Table 1:** Characteristics of patients included in this study

Characteristic	All data (n = 124)		*p* value
	GIST (n = 90)	Others (n = 34)	
Tumor size*	4.35 ± 2.87	3.87 ± 3.35	0.484
Long/short diameter*	1.26 ± 0.29	1.52 ± 0.61	0.032
CT value*	34.18 ± 5.69	36.79 ± 9.97	0.192
Arterial phase enhancement**	14.60 ± 13.29	17.00 ± 13.38	0.800
Venous phase enhancement**	34.15 ± 16.63	35.85 ± 19.91	0.423
Homogeneous enhancement*			
Yes	46	22	0.218
No	33	8	
Bleeding^#^			
Yes	9	3	1.000
No	66	28	
Ulcer on the surface^#^			
Yes	12	3	0.545
No	63	28	
Calcification inside^##^			
Yes	7	4	0.734
No	33	15	
Liquid area inside^##^			
Yes	12	0	0.006
No	28	19	
White blood cell (× 10^9^/L)^+^	5.74 ± 2.14	5.55 ± 1.43	0.645
Neutrophil (× 10^9^/L)^+^	3.49 ± 1.93	3.24 ± 1.33	0.475
Lymphocyte (× 10^9^/L)^+^	1.62 ± 0.52	1.67 ± 0.57	0.616
Platelet (× 10^9^/L)^+^	219.5 ± 56.50	228.00 ± 81.77	0.517
Haemoglobin (g/L)^+^	129.77 ± 23.78	128.29 ± 18.17	0.745
ALT^+^	21.18 ± 10.86	17.09 ± 8.08	0.026
AST^+^	22.78 ± 10.79	20.03 ± 7.30	0.173
Albmin (g/L)^+^	40.25 ± 3.87	40.76 ± 3.53	0.501
Fibrinogen^+^	302.36 ± 56.55	303.50 ± 70.41	0.925
De Ritis ratio^++^	1.21 ± 0.44	1.30 ± 0.52	0.324
PNI^++^	48.33 ± 5.12	49.12 ± 4.34	0.428
SII^++^	527.31 ± 394.00	518.67 ± 400.14	0.914
PLR^++^	147.67 ± 57.70	155.44 ± 93.01	0.577
NLR^++^	2.42 ± 1.73	2.29 ± 1.65	0.712

PNI = serum albumin (g/L) + 0.005 × lymphocyte count (/mm^3^)

SII = Platelet (counts/L) × Neutrophils (counts/L)/Lymphocytes (counts/L)

De Ritis ratio = ALT/AST

*ALT* alanine transaminase, *AST* glutamic oxaloacetic transaminase, *PNI* prognostic nutritional index, *SII* systemic immune-inflammation index, *PLR* platelet–lymphocyte ratio, *NLR* neutrophillymphocyte ratio

*Tumor performance under CT scan

**Tumor performance under enhanced CT scan

^#^Tumor performance under endoscopy

^##^Tumor performance under EUS

^+^Counts in peripheral blood

^++^Calculated by indicators in peripheral blood

### Predictor selection

**Fig. 1:**
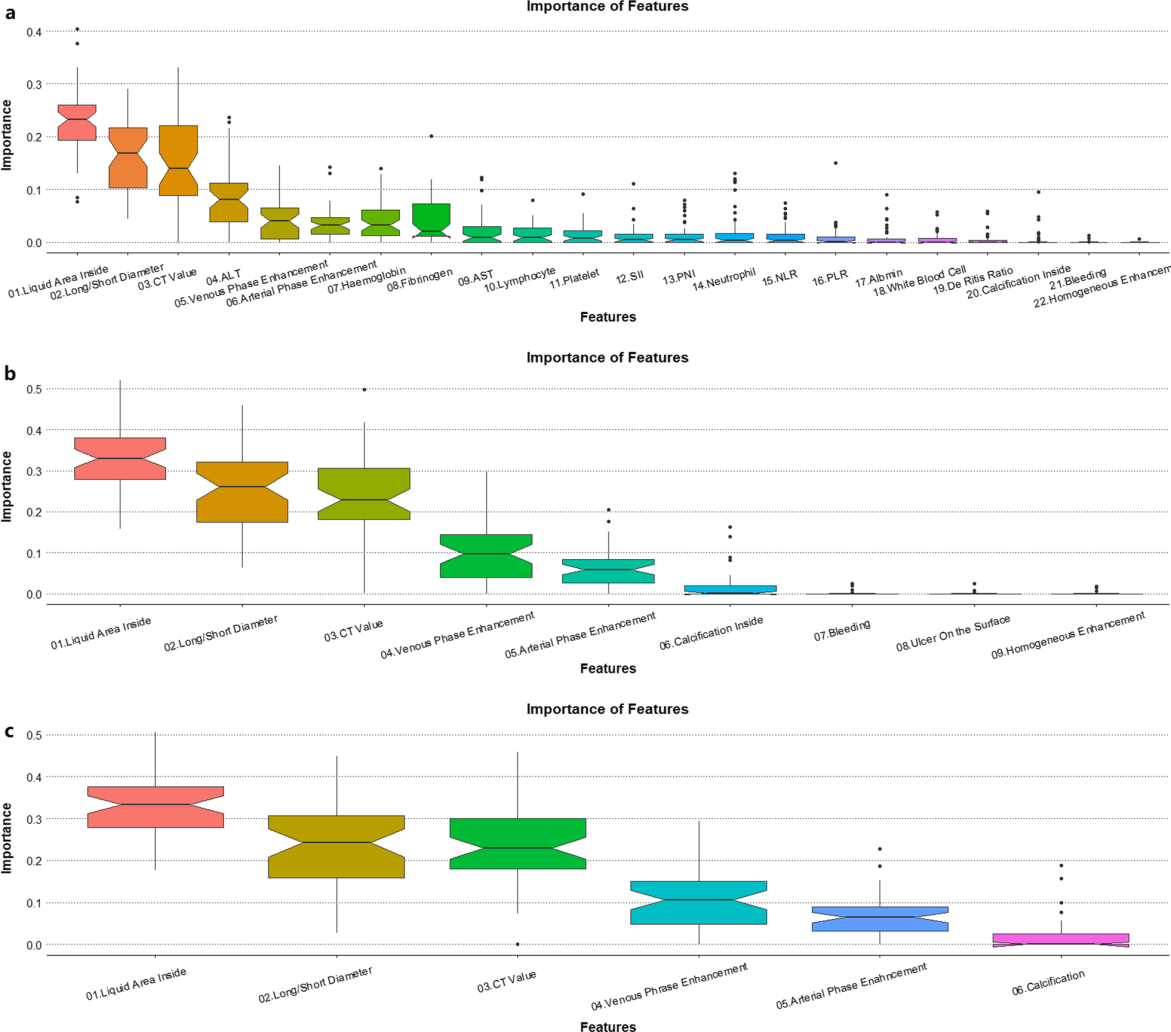
The process of predictor selection. In the initial XGBoost model, we used all clinical data before surgery to construct the model. **a** Shows the importance of each feature. It’s easy to find out that most hematological test features don’t have an important impact on GIST diagnosis except ALT. Similarly, we built the second model using the data only from enhanced CT, endoscopy and EUS (**b**). The latter three features also have little importance, and that’s why they were excluded. The first 6 predictors were determined to be the most important features and utilized in the final model development. The importance of the six predictors in the final XGBoost model are shown in **c**. The most important predictors of this model is the existence of liquid area inside the tumor under EUS, following by the ratio of long and short diameter under CT, the CT value of the tumor, the enhancement of the tumor in arterial period and venous period, existence calcified area inside the tumor under EUS. (The specific values and 95 CI of importance are shown in Additional file 2: Table S1.)

We can see the importance of each factor in the two models constructed at the beginning of this study from Fig. 1. We used all 22 factors for the first model development, and we can find that most of the hematological indicators except ALT have little effect on the diagnosis of GIST (Fig. 1a). Therefore, the second model was conducted after all hematological test data were excluded. Similarly, we can see the importance of each factor in the second model in Fig. 1b. It is not difficult to find out that the latter three factors have little impact on the diagnosis of GIST, so they were also excluded. The remaining six predictors have an important impact on the diagnosis of GIST, including the ratio of long and short diameter under CT, the CT value of the tumor, the enhancement of the tumor in arterial period and venous period, existence of liquid area and calcific area inside the tumor under EUS. The correlation between most of the selected predictors are not strong (Additional file 1: Fig. S1).

### Model performance

The optimal cut-off value of this model is 0.684. That is, when the output calculation result is higher than 0.684, the patient would be diagnosed as “GIST”, and when the output calculation result is lower than 0.684, it indicates “Other SMT”. According to this cut-off value, the performance of the model in the validation dataset is calculated (Table 2): the auROC value is 0.77 (0.57–0.90), the C-index is 0.76 (0.56–0.89). The accuracy of model prediction in the validation dataset is 0.73 (0.58–0.88), precision is 0.79 (0.60–0.95), recall is 0.87 (0.67–1.00), and f1-score is 0.82 (0.70–0.92).

**Table 2:** Validation performance of the model

Metric	Performance (95 CI)
Accuracy	0.73 (0.58–0.88)
Precision	0.79 (0.60–0.95)
Recall	0.87 (0.67–1.00)
f1-score	0.82 (0.70–0.92)
auROC	0.77 (0.57–0.90)
C-index	0.76 (0.56–0.89)

Accuracy: number of correct predictions/total number of all predictions

Precision: number of correct positive predictions/number of positive predictions

Recall: number of correct positive predictions/number of all positive individuals

F1-score: the harmonic mean of precision and recall

auROC: area under the receiver operating characteristic curve

C-index: concordance index

### Predictors importance

**Fig. 2:**
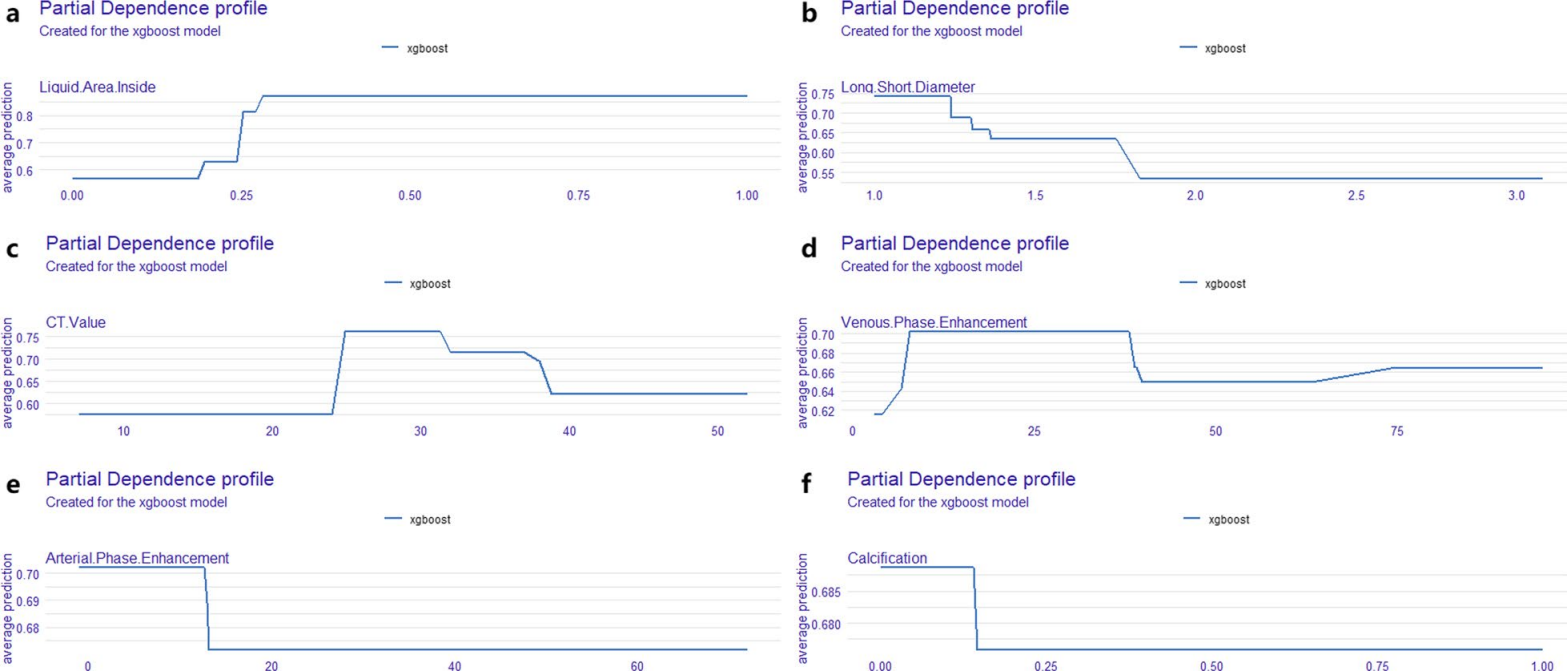
Partial importance of features in the final model. Round or round-like tumors (**b**) with a CT value of around 30 (25–37) (**c**) and delayed enhancement (**d**, **e**), as well as liquid (**a**) but not calcified (**f**) area inside the tumor best indicate the diagnosis of GIST

The most important predictors of this model is the existence of liquid area inside the tumor under EUS, following by the ratio of long and short diameter under CT, the CT value of the tumor, the enhancement of the tumor in arterial period and venous period, existence calcific area inside the tumor under EUS (Fig. 1c; Additional file 2: Table S1). The influence of each predictor on the predicted outcome is shown in Fig. 2a–f. Round or round-like tumors with a CT value of around 30 (25–37) and delayed enhancement, as well as liquid but not calcific area inside the tumor best indicate the diagnosis of GIST.

### Prediction and interpretation at the individual scale

**Fig. 3:**
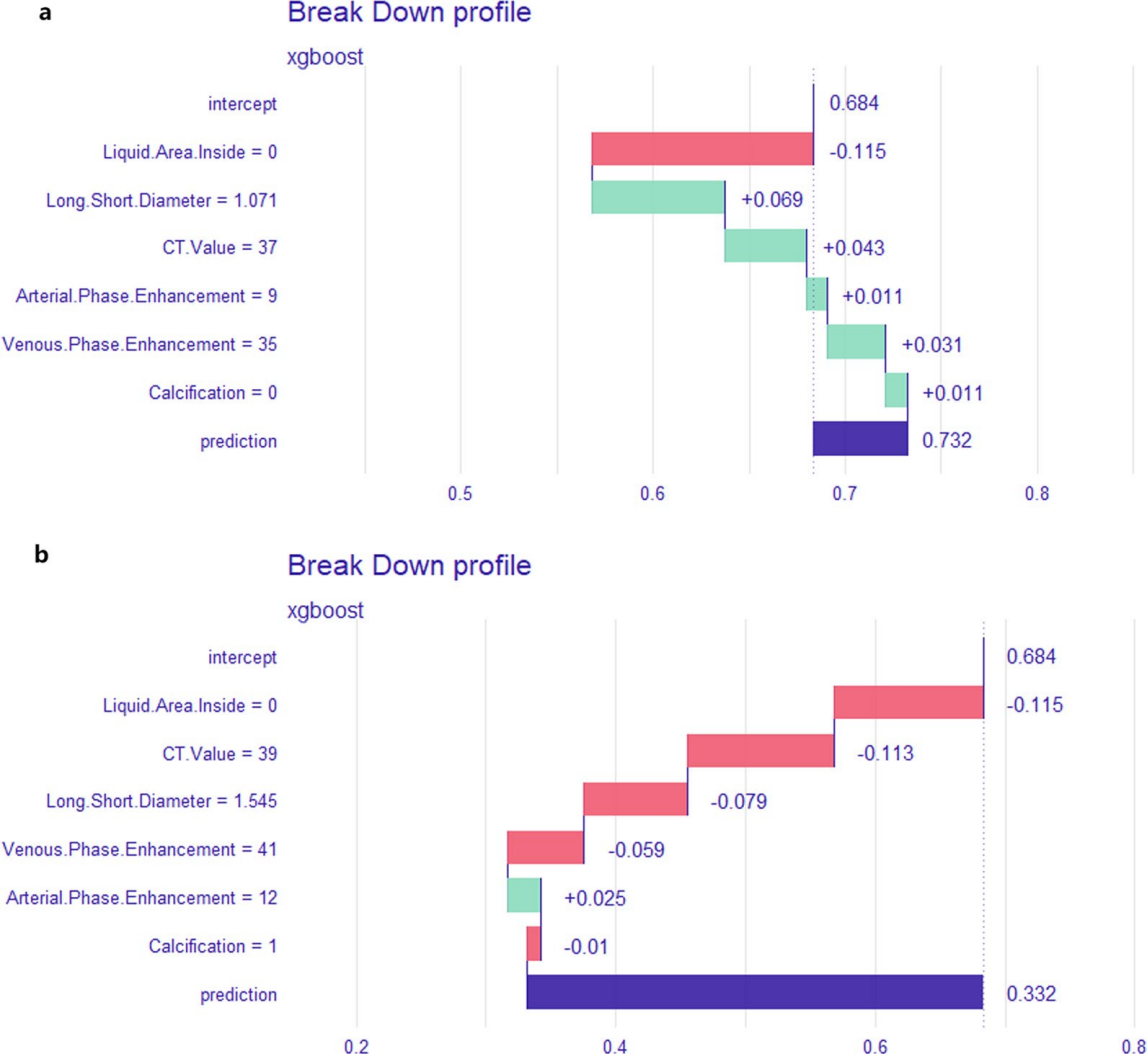
Prediction in individual scale. Inputting the data of two patients into this model yielded individualized results. The length of the square indicates the impact on the outcome. Green indicates “GIST”, while red indicates “Other SMT”. The final prediction result is compared with 0.684. A prediction value higher than 0.684 is predicted as “GIST”, while lower than 0.684 is diagnosed as “Other SMT”. The prediction result of patient 1 (**a**) in this model is “GIST”, and patient 2 (**b**) is “Other SMT”

We input the clinical data of 2 patients into this model. The CT value of Patient 1's tumor was 37, and the ratio of long to short diameter of the tumor was 1.071. The CT value in arterial phase increased by 9 while 35 in venous phase. EUS showed that the tumor did not contain a liquid or calcific area. After inputting all these data into the model, the prediction value of patient 1 was 0.732, which was higher than 0.684, so the predictive result of patient 1 in this model is “GIST” (Fig. 3a). The CT value of Patient 2's tumor was 39, increased by 12 in arterial phase and 41 in venous phase. The ratio of long to short diameter of the tumor was 1.545. No liquid area but some calcification were found under EUS. After inputting these data into the model, the prediction value of patient 2 was 0.332, which was lower than 0.684, and the prediction result of patient 2 was “Other SMT” (Fig. 3b).

## Discussion

We have developed and validated a novel diagnostic model for gastric GISTs by an XGBoost machine based on a single-center retrospective dataset. The predictors selected into this study through initial XGBoost model include: the ratio of long and short diameter under CT, the CT value of the tumor, the enhancement of the tumor in arterial period and venous period, existence of liquid area and calcific area inside the tumor under EUS. Considering that we only included the patients who were initially diagnosed GIST and excluded those with other SMTs that can be easily recognized in CT scan or endoscopy at their first presentation, the model yielded satisfactory result for validation and provided a novel measure for those patients with other SMTs that were extremely similar with GIST in CT and endoscopic features.

Current guidelines around the world recommend enhanced CT scan, endoscopy and EUS as the primary diagnostic modalities, while determining whether a tumor is GIST still relies on EUS or CT guided fine-needle aspiration biopsy [5]. Although the guidelines recommend that fine-needle aspiration biopsy should be performed for those considering GISTs, preoperative biopsy is not promoted widely in some countries or regions in the world due to poor conditions or some other reasons and guidelines also mention that preoperative biopsy can be 'omitted' or 'not necessary' for limited resectable SMTs [11, 12]. In addition, fine needle aspiration biopsy may give false negative results due to its small specimen size [5]. Therefore, although this invasive test has been proven secure and will not result in tumor rupture or GI-tract dissemination, the clinical application rate is still very low (2/124 in our database). For patients without preoperative biopsy result, the misdiagnosis rate is rather high (34/122 in our database), which is proof that the use of enhanced CT scan, endoscopy or EUS alone to diagnose GIST is not accurate enough [3, 4].

Recently, many studies demonstrated the influence of peripheral blood hematological indicators of systemic inflammation or nutrition on the long-term prognosis of various cancers and even GISTs after surgical resection [9, 13–16]. It’s suggested that higher-risk tumors, including GISTs, have a stronger impact on the patients’ nutritional state and inflammatory levels. Compared with other benign or low-malignant gastrointestinal SMTs, GISTs should further decrease nutritional indicators and increase inflammatory indicators. Therefore, we included peripheral blood systemic inflammation and nutrition indicators in our initial analysis. But not surprisingly, we found that these hematological test data, except for ALT, have little effect on the outcome. As is currently no evidence to support the impact of ALT level changes alone on the diagnosis of GIST, we excluded all hematological test data in the next model development.

Recent years witnessed the boost in artificial intelligence application in the medical field, assisting in disease detection, diagnosis and treatment decision-making [17]. As the concept of precision medicine being promoted for years, the use of machine learning algorithms to help clinical diagnosis and treatment has become an inevitable trend. However, data science is not able to perfectly match the facts all the time. Selecting appropriate machine learning algorithm is crucial to yielding meaningful and useful results, yet it is not an easy process [18]. Ensemble-based classifier is better than any single classifier in analyzing the influence of the combination of various factors on outcome. In terms of complex nonlinear multi-feature models such as predictive clinical models, the tree-boosting machine has better performance, giving both the importance and ranking of each factor simultaneously [19, 20]. The XGBoost algorithm has been applied in various clinical studies in constructing disease prediction models, and proved to have good validation results [21, 22]. Therefore, it is logical to choose XGBoost in our model development.

The first and foremost achievement of this research is the development of a GIST clinical diagnostic model. All patients included in our dataset were initially diagnosed as gastric GIST after preoperative examinations, and the model proved to have satisfactory validation results in such circumstance. The model outputs the importance of each predictor, suggesting that the existence of liquid area inside the tumor under EUS is the most important predictor, followed by the ratio of long and short diameter under CT, the CT value of the tumor, the enhancement of the tumor in arterial period and venous period, existence calcific area inside the tumor under EUS. All the data we used to develop the model came from the patients’ preoperative clinical examinations and hematological tests, which would not cause any additional pain or economic stress for the patients.

The main limitation of this study is that it is a single center, small sample, retrospective study, with 124 patients included. Large-scale, multi-center studies are required for the development of more accurate models. In addition, it should be noted that the analytic process using the gradient-boosting machine in this study was entirely based on data science. Clinical results may be different from mathematical calculation. At present, there is no perfect and absolutely accurate statistical algorithm that can predict the exact clinical outcome of every patient. Moreover, this model is only suitable for patients who consider gastric GIST as their initial clinical diagnosis and cannot perform biopsy for some reason before surgery. For patients who are not considered GIST initially or have a SMT out of stomach, this model may yield inaccurate prediction, or even contrary results. This model is only a tool to assist clinical diagnosis, by giving an interpretation of the clinical test results to doctors, aiding them in making the final diagnosis and intervention measures. In the future, nationwide, multi-center large scale studies are expected for further improvement of current models.

## Supplementary Information

**Additional file 1: Supplementary Figure 1.** Correlation of the 6 features included in the final model.

**Additional file 2: Supplementary Table 1.** Feature importance in the final XGBoost model.

## Data Availability

We uploaded the R code of our study on the website: https://github.com/hbz0411/GIST_diagnose for the purpose of academic sharing. The datasets analyzed during this study are available from the corresponding authors on reasonable request.
