# Supplementary material for: Development and validation of a novel diagnostic model for initially clinical diagnosed gastrointestinal stromal tumors using an extreme gradient-boosting machine
Source: BMC Gastroenterol. 2021 Dec 18;21:481. doi: 10.1186/s12876-021-02048-1 (PMC8684147; doi:10.1186/s12876-021-02048-1)
Supplement: Supplementary file 1 — Additional file 1: Supplementary Figure 1. Correlation of the 6 features included in the final model. [file 12876_2021_2048_MOESM1_ESM.docx]

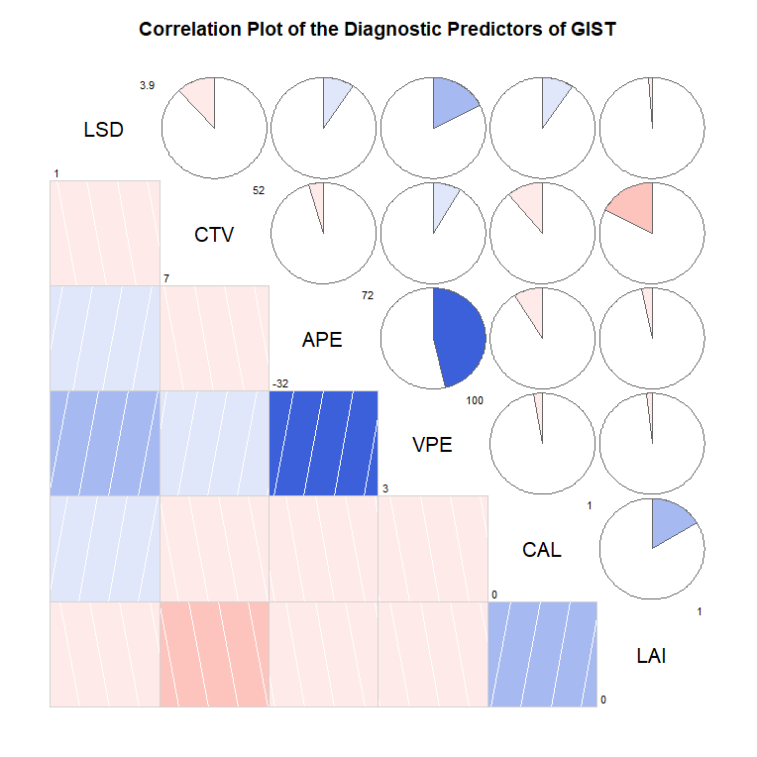


**Supplementary Fig 1. Correlation of the 6 features included in the final model.**

LSD, long/short diameter of the tumor; CTV, CT value of the tumor; APE, arterial phase enhancement; VPE, venous phase enhancement; CAL, Calcification inside the tumor; LAI, liquid area inside the tumor.

Blue indicates positive correlation. Red indicates negative correlation. The darker the color, the stronger the correlation. The pie chart indicates the percentage of correlation. The diagonal line is the feature name, and the number in it indicates the value range of the feature.

We can easily find out that most features don’t have a strong correlation except APE and VPE.
