# Supplementary material for: Development and validation of a novel diagnostic model for initially clinical diagnosed gastrointestinal stromal tumors using an extreme gradient-boosting machine
Source: BMC Gastroenterol. 2021 Dec 18;21:481. doi: 10.1186/s12876-021-02048-1 (PMC8684147; doi:10.1186/s12876-021-02048-1)
Supplement: Supplementary file 2 — Additional file 2: Supplementary Table 1. Feature importance in the final XGBoost model. [file 12876_2021_2048_MOESM2_ESM.docx]

| **Feature** | **Importance (95 CI)** | |
| --- | --- | --- |
| **Liquid area inside the tumor** | | 0.336 (0.197-0.460) |
| **Long/short diameter of the tumor** | | 0.241 (0.081-0.393) |
| **CT value of the tumor** | | 0.231 (0.076–0.389) |
| **Venous phase enhancement** | | 0.102 (0.000-0.263) |
| **Arterial phase enhancement** | | 0.061 (0.000-0.187) |
| **Calcification inside the tumor** | | 0.009 (0.000-0.157) |

**Supplementary Table 1. Feature importance in the final XGBoost model.**

The distribution (95 CI) of importance of each predictor was calculated by repeating the process of random grouping and model building 200 times and reporting the 2.5 and 97.5 percentiles. The box plot of this table were shown in Fig 1c.
